# Association of cardiac and vascular changes with ambient PM_2.5 _in diabetic individuals

**DOI:** 10.1186/1743-8977-7-14

**Published:** 2010-06-02

**Authors:** Alexandra Schneider, Lucas M Neas, Don W Graff, Margaret C Herbst, Wayne E Cascio, Mike T Schmitt, John B Buse, Annette Peters, Robert B Devlin

**Affiliations:** 1 Helmholtz Zentrum München, German Research Center for Environmental Health, Institute of Epidemiology, Neuherberg, Germany; 2 Environmental Public Health Division, National Health and Environmental Effects Research Laboratory, Environmental Protection Agency, RTP, North Carolina, USA; 3 Celerion, Lincoln, Nebraska, USA; 4 University of North Carolina, School of Medicine, Chapel Hill, North Carolina, USA; 5 Brody School of Medicine, and the East Carolina Heart Institute at East Carolina University, Greenville, North Carolina, USA; 6 Focus Network Nanoparticles and Health (NanoHealth), Helmholtz Zentrum München, German Research Center for Environmental Health, Neuherberg, Germany

## Abstract

**Background and Objective:**

Exposure to fine airborne particles (PM_2.5_) has been shown to be responsible for cardiovascular and hematological effects, especially in older people with cardiovascular disease. Some epidemiological studies suggest that individuals with diabetes may be a particularly susceptible population. This study examined effects of short-term exposures to ambient PM_2.5 _on markers of systemic inflammation, coagulation, autonomic control of heart rate, and repolarization in 22 adults (mean age: 61 years) with type 2 diabetes.

**Methods:**

Each individual was studied for four consecutive days with daily assessments of plasma levels of blood markers. Cardiac rhythm and electrocardiographic parameters were examined at rest and with 24-hour ambulatory ECG monitors. PM_2.5 _and meteorological data were measured daily on the rooftop of the patient exam site. Data were analyzed with models adjusting for season, weekday, meteorology, and a random intercept. To identify susceptible subgroups, effect modification was analyzed by clinical characteristics associated with insulin resistance as well as with oxidative stress and by medication intake.

**Results:**

Interleukin (IL)-6 and tumor necrosis factor alpha showed a significant increase with a lag of two days (percent change of mean level: 20.2% with 95%-confidence interval [6.4; 34.1] and 13.1% [1.9; 24.4], respectively) in association with an increase of 10 μg/m^3 ^in PM_2.5_. Obese participants as well as individuals with elevated glycosylated hemoglobin, lower adiponectin, higher ferritin or with glutathione S-transferase M1 null genotype showed higher IL-6 effects. Changes in repolarization were found immediately as well as up to four days after exposure in individuals without treatment with a beta-adrenergic receptor blocker.

**Conclusions:**

Exposure to elevated levels of PM_2.5 _alters ventricular repolarization and thus may increase myocardial vulnerability to arrhythmias. Exposure to PM_2.5 _also increases systemic inflammation. Characteristics associated with insulin resistance or with oxidative stress were shown to enhance the association.

## Background

As a consequence of life-style patterns contributing to obesity the number of people with diabetes in the United States has reached 26.8 million in 2010 and by 2030 is expected to affect 12% of adults (age: 20-79 years) as compared to 10.3% today; worldwide 285 million people have type 2 diabetes and by 2030, that number is expected to rise to 439 million [1]. Metabolic abnormalities caused by diabetes induce vascular dysfunction that predisposes diabetic individuals to atherosclerosis and its complications [2]. The risk of developing coronary, cerebrovascular and peripheral vascular disease is increased up to four-fold [2]. In addition, diabetic individuals have greater disease progression and higher cardiovascular mortality rates compared to non-diabetic individuals [3].

Individuals having diabetes have been shown to be at greater risk for morbidity and mortality associated with exposure to ambient air pollution particulate matter (PM) [4,5]. Although many people with diabetes have secondary cardiovascular complications, as a group they show larger responses to PM than people with cardiovascular (CV) disease and no diabetes [6]. However, the mechanisms accounting for the increased risk is unknown.

Epidemiological as well as toxicological research have shown PM-induced changes in autonomic nervous system function [7-10] as well as in vascular factors involved in inflammation and coagulation [11-16], particularly in elderly people with CV disease. Since diabetic individuals appear to have additional risk factors for PM-associated mortality and morbidity beyond those found in people with CV, we hypothesize that changes in these health endpoints might be even more pronounced in people with diabetes than in people with CV disease without diabetes, who have been characterized in other studies.

In this study we assessed the effects of ambient fine particles (PM_2.5_) on repeated measures of cardiac autonomic balance, systemic inflammation and coagulation in individuals with type 2 diabetes. Fluctuations in daily air pollution measurements from a local air monitoring station were associated with changes in health effect parameters which provide information about responses by the immune, vascular, cardiac, hematologic, and autonomic nervous systems. To determine if severity of diabetes was associated with increased responsiveness to PM, we analyzed effect modifications associated with insulin resistance (obesity, age, poor glycemic control, adiponectin). We also analyzed effect modifications associated with markers of oxidative stress or potential markers of susceptibility to oxidative stress (myeloperoxidase (MPO), ferritin and glutathione S-transferase M1 (*GSTM1*)), and medication usage (beta-blockers, statins, and aspirin).

## Materials and methods

### Study population

Twenty-two volunteers, aged 48-78, with type 2 diabetes were identified through the University of North Carolina (UNC) Diabetes Clinic and through newspaper advertisements. Details on the inclusion and exclusion criteria have been previously published [17]. Potential participants had to have a diagnosis of type 2 diabetes, but without taking insulin; and be on a stable medication regimen throughout their participation. Volunteers were excluded if they had a recent vascular event or intervention such as coronary artery graft bypass surgery or percutaneous coronary intervention. Participants were asked to refrain from vigorous exercise on study-mornings and to refrain from medications like anti-oxidants (vitamins C and E), fish oil, niacin, arginine, over-the-counter vasoactive agents such as decongestants, anti-inflammatory agents such as ibuprofen, naproxen or aspirin unless it was prescribed as a daily medication (in which case it was continued) for the week prior to the study as well as the week of the study. Participants were also asked to refrain from use of phosphodiesterase enzyme inhibitors during the week of the study.

Each participant visited the U.S. Environmental Protection Agency's National Health Effects Environmental Laboratory, Environmental Public Health Division (EPHD) in Chapel Hill, NC, on five consecutive weekdays between November 2004 and December 2005. Data on health status, pulmonary and cardiac symptoms, medication, and smoking history were obtained at baseline and during four follow-up visits. Altogether a maximum of 88 observations were available for analysis, depending on the examined health outcome. All volunteers signed a written consent form and the study protocol was approved by the UNC Human Studies Biomedical Institutional Review Board as well as by the EPA.

### Clinical measurements

#### Clinical procedures

On Monday morning of each examination week the participants completed a baseline questionnaire. In each of the next four mornings, participants checked into the medical station in a fasting state and without having taken their anti-diabetic medication. Upon arriving at the medical station each morning, fasting glucose concentration was measured with a point of care glucose meter.

#### Venipuncture and assays

Each morning approximately 60 ml of peripheral venous blood of was taken from an antecubital site. Both serum and plasma were obtained and stored at -80°C. Differential cell counts, clinical chemistry endpoints, and inflammation and coagulation markers were selected because previous studies suggested they may change in association with PM (Table 1).

**Table 1:** Hematological measurements.

*Inflammation/Coagulation*	
Acute-phase reaction	C-reactive protein (CRP), albumin, fibrinogen
Pro-inflammatory cytokines	Interleukin-6 (IL-6), tumor necrosis factor alpha (TNFα)
Endothelial dysfunction	Soluble intercellular adhesion molecules-1 (sICAM-1), soluble vascular cell adhesion molecules-1 (sVCAM-1), soluble endothelial-leukocyte adhesion molecule (E-selectin)
Platelet activation	Von Willebrand factor (vWf)
Coagulation pathway	Factor VII, factor IX, protein C, fibrinogen
Fibrinolysis pathway	Plasminogen, D-dimer, tissue plasminogen activator (t-PA), plasminogen activator inhibitor (PAI-1)

*Blood panel*	

Blood lipids	Total cholesterol (TC), triglycerides, very low-density lipoprotein (VLDL), low-density lipoprotein (LDL), high-density lipoprotein (HDL)
Red blood cells count (RBC)	Mean corpuscular volume (MCV)
White blood cells count (WBC)	Monocytes, neutrophils, eosinophils, lymphocytes → absolute number and percentage of WBC
Platelets	
Total hemoglobin	
Mean corpuscular hemoglobin (MCH)	

Basic blood chemistry, blood lipids, ferritin, glycosylated hemoglobin A1c (HbA1c), homocysteine, albumin and complete blood counts with differential were measured and calculated by LabCorp (Burlington, NC) on the day the blood was drawn. HbA1c was only measured from the first blood sample. C-reactive protein (CRP) levels were quantified using a commercially available enzyme-linked immunosorbent assays (ELISA) kit purchased from Alpco Diagnostics (Salem, NH). Paired antibodies were purchased from Enzyme Research Labs (South Bend, IN) and used to develop ELISA assays to quantify levels of fibrinogen, protein C, factor VII, factor IX, plasminogen, and tissue plasminogen activator (t-PA). Similarly, ELISA assays were developed using paired antibodies for D-dimers and von Willebrand factor (vWf) purchased from Diagnostica Stago (Asnieres-Sur-Seine, France). Myeloperoxidase (MPO), soluble intercellular adhesion molecule (s-ICAM)-1, soluble vascular cell adhesion molecule (s-VCAM)-1, soluble endothelial-leukocyte adhesion molecule (E-selectin), plasminogen activator inhibitor (PAI)-1, tumor necrosis factor alpha (TNFα), and interleukin (IL)-6 were quantified using commercially available ELISA kits purchased from Lincoplex (Linco Research, Inc., St. Charles, MO) and run on a Luminex 100 Multiplex system. Adiponectin levels were measured using a commercially available kit purchased from R&D Systems (Minneapolis, MN).

Regarding genetics, we measured the null-polymorphism of glutathione S-transferase M1 (*GSTM1*), a gene that belongs to the antioxidant defense family. DNA was isolated from white blood cells using the QIAamp DNA Mini Kit (Qiagen Inc., Valencia, CA) following the Blood and Body Fluid Spin Protocol (Qiagen). The *GSTM1 *genotypes were determined using real-time PCR by TaqMan 7500 (Applied Biosystems, Foster City, CA). The forward/reverse primers, probe and real time PCR conditions were adapted from Gilliland et al (2002) [18]. All samples were also analyzed with primers and probes for the human actin gene to verify the presence of amplifiable DNA.

After the blood sample was taken, a resting systolic (BPsys) and diastolic (BPdia) blood pressure was taken (Table 2).

**Table 2:** Electrocardiogram and blood pressure parameters.

*Heart rate variability (HRV) (24 h): time domain*	
RMSSD	Root mean square of successive differences
SDNN	Standard deviation of NN (normal-to-normal)
pNN50	Percentage of adjacent NN-intervals which differ more than 50 ms

*Heart rate variability (HRV) (5 min at rest): frequency domain*	

HR	Heart rate
TP	Total power (approximately = 0.40 Hz)
LF^a^	Low frequency band (0.04-0.15 Hz)
HF^a^	High frequency band (0.15-0.40 Hz)
LF/HF	Low-to-high frequency power ratio

*Repolarization events (5 min at rest)*	

QTc	Bazett-corrected QT interval (correction: QT/sqrt(NN))
QT norm	Normalized QT variance
QTVI	QT variability index
T wave amplitude	Amplitude of T wave
T wave complexity	Principal component analysis of T wave
Variability of T wave complexity	Standard deviation of T wave complexity

*Blood pressure*	

BPsys	Systolic blood pressure
BPdia	Diastolic blood pressure

^a^Represented as absolute values of power (unit: ms^2^) and as the relative value of each power component in proportion to total power minus the very low frequency component (unit: normalized units)

#### ECG measurements

Continuous ambulatory electrocardiograms (Holter ECGs) were collected for three 24-hour slices using a Mortara H12+ 12-Lead ECG Recorder (Mortara Instrument Co., Milwaukee, WI) sampling at 180 Hz. Electrodes were changed each morning to ensure adequate skin contact and new batteries and a fresh flash card were used. A trained research nurse manually edited the sequence of ECG complexes to ensure proper labeling of each QRS complex. RR-intervals that were more or less than 20% of the previous RR-interval were defined as abnormally long or short intervals and were interpolated using Mortara algorithms. Subsequent heart rate variability (HRV) indices in both the time and frequency domains were calculated from these edited records (Table 2). A detailed description of the measurements has been previously published [19]. Data was collected during a 30-minute period each morning while the subjects rested quietly in a darkened room.

Time-domain parameters were calculated over a 24-hour period starting at the beginning of the resting period. The standard deviation of normal-to-normal intervals (SDNN), root-mean-square of successive differences (RMSSD) and percentage of adjacent normal-to-normal intervals which differ more than 50 ms (pNN50) were assessed. A 5-minute segment during the end of the resting period was used to calculate frequency domain and repolarization indices. From the spectra the frequency bands 0.04-0.15 Hz (low frequency (LF)) and 0.15-0.40 Hz (high frequency (HF)) were calculated in absolute and heart rate-normalized units (LF_norm and HF_norm). Total power (TP), the low-to-high frequency power ratio (LF/HF) and resting heart rate (HR) were also determined.

Because spatiotemporal heterogeneity of repolarization contributes to the initiation of arrhythmia, we also calculated markers of cardiac repolarization including heart rate (Bazett)-corrected QT interval (QTc), normalized QT variance (QT norm), QT variability index (QTVI), T wave amplitude, and T wave complexity together with the variability (standard deviation) of T wave complexity. QTc is reported as the average of eight consecutive beats on a composite of all leads. The normalized QT variance was calculated by QT_norm = QT_variance/(mean QT)^2^. The QT variability index (QTVI) was derived for each interval according to the equation: QTVI = log_10 _[(QT_v_/QT_m_^2^)/(HR_v_/HR_m_^2^)] [20] with the heart rate mean (HR_m_) and variance (HR_v_) and QT interval mean (QT_m_) and variance (QT_v_) being computed from the respective time series for each 5-minute interval. For T wave amplitude, original ECG leads I, II, V1 to V6 were used and the median value from those eight original leads was taken for each cardiac cycle and averaged over five minutes. T wave complexity was measured in each beat by principal component analysis (PCA) based on all 12 leads and averaged over the 5-minute period.

Altogether, four blood samples, four blood pressure measurements, four 5-minute resting ECGs, and three 24-hour ECGs were intended to be collected from each subject.

### Air pollution monitoring

Concentrations of fine ambient PM (average from 9 AM - 9 AM) were measured on each clinic day and for the four prior days with a 3000K Versatile Air Pollution Sampler (URG Corp., Chapel Hill, NC) [21] located on the EPHD rooftop approximately 30 meters above ground level. In addition, daily 24-hour concentrations (midnight to midnight) of fine ambient PM_2.5 _mass were obtained from a monitoring station located approximately 44 kilometers (27 miles) east of the EPHD and operated by the State of North Carolina. The Spearman correlation between both PM_2.5 _measurements was 0.85 across the 14 month study period and the network data was used to impute missing rooftop data based on a linear regression model. Of thirteen daily averages relevant for the health outcome analysis, eleven could be imputed and only two remained missing.

Continuous 2-minute measurements of air temperature, barometric pressure and relative humidity were obtained from the EPHD rooftop and 24-hour averages were calculated. Rooftop data was complete for the health outcome analysis, so no imputation was necessary.

### Statistical analyses

The study was conducted as a panel study with four (three for 24-hour ECGs) repeated measurements per participant. Thus every person acted as his/her own control which limited the need for an adjustment for patient characteristics in the analysis.

Data were analyzed using the statistical package SAS Version 9.1 (SAS Institute, Inc., Cary, NC). For the analysis of the PM effect, additive mixed models with a random participant effect and "compound symmetry" covariance structure were used [22,23]. Model building to identify meteorological and temporal determinants, as potential confounders, was done for each outcome variable separately. Model fit was assessed by the Akaike Information Criterion (AIC). Details on the confounder selection process can be found in Schneider et al. (2008) [17].

The exposure, in this case rooftop PM_2.5 _mass, was considered as an immediate (lag 0) or a delayed (lag 1 to lag 4) linear effect. Effect estimates are presented as percentage changes of the mean outcome variable together with 95%-confidence intervals (CI) for a 10 μg/m^3 ^increment in ambient PM_2.5 _mass.

Sensitivity analyses were done by changing the covariance structure to first order autocorrelation as well as by repeating parts of the analysis with PM_2.5 _values that were not imputed. In addition, analyses were repeated using network PM_2.5 _data imputed with rooftop PM_2.5 _data as exposure. Individuals that had experienced PM_2.5 _values exceeding 35 μg/m^3 ^during their relevant days of exposure were excluded to examine if health effects occurred also below current 24-hour U.S. National Ambient Air Quality Standard (NAAQS). Moreover, to assess the heterogeneity across study participants in this repeated measures study, we examined patient-specific, random slope-models for selected PM_2.5 _results.

Effect modification was examined using dichotomous indicator variables (Table 3). For blood endpoints, we considered body mass index (BMI) (cut point 30 kg/m^2^), age (cut point 60 years), HbA1c (cut point 7%, measured only once), adiponectin (cut point 3700 ng/ml, mean of repeated measurements per individual used for grouping), MPO (cut point 7 ng/ml, daily level used for grouping), ferritin (cut point 60 ng/ml, daily level used for grouping), and statins intake (yes/no). In addition, effect modification by aspirin intake (yes/no) was examined for coagulation markers fibrinogen, factor VII, factor IX, and protein C. For ECG endpoints, we considered beta-blockers intake (yes/no). Cut points were typically near the median value seen in the 22 participants. In addition, gene-environment interaction was assessed for the null-polymorphism of *GSTM1*.

**Table 3:** Potential selected effect modifiers.

*Clinical characteristics associated with insulin resistance*	
Body mass index (BMI)	Cutpoint 30 kg/m^2^
Age	Cutpoint 60 years
Glycosylated hemoglobin A1c (HbA1c)	Cutpoint 7%
Adiponectin-level in blood	Cutpoint 3700 ng/ml

*Clinical characteristics associated with oxidative stress*	

Myeloperoxidase (MPO)-level in blood	Cutpoint 7 ng/ml
Ferritin-level in blood	Cutpoint 60 ng/ml
Glutathione S-transferase M1 (*GSTM1*) null polymorphism	Yes/No

*Medication intake*	

Beta-adrenergic receptor (BB)	Yes/No
Statins	Yes/No
Aspirin	Yes/No

## Results

### Participant characteristics

The participants' age ranged between 48 and 78 years, and almost two thirds of the recruited volunteers were male. All participants were current non-smokers, but almost half of them were ex-smokers. Their BMIs ranged between 20 and 44 kg/m^2 ^with 41% (n = 9) of the participants being overweight (20-25 kg/m^2^) and 55% (n = 12) being obese (≥ 30 kg/m^2^) (Table 4). Less than 50% had a systolic blood pressure greater than 140 mmHg indicating stage 1 hypertension. Duration of type 2 diabetes ranged from two months to 23 years (Table 5). Most participants had a history of hypertension and dyslipidemia. The majority was treated with oral anti-hyperglycemic medications and half of them were taking statins and anti-hypertensives. About two-thirds of the participants regularly took aspirin.

**Table 4:** Description of the study population characteristics: current non-smoking subjects with type 2 diabetes mellitus.

Individual Characteristics	N = 22 individuals
	Mean (± SD) or total number N (%)
Age [yrs]	61 (± 8)
Age ≥ 60 yrs	13 (59)
Gender: male	14 (64)
Ethnicity:	
Caucasian	15 (68)
African-American	6 (27)
Hispanic-American	1 (5)
Body mass index (BMI) [kg/m^2^]	33 (± 7)
Body mass index (BMI) ≥ 30 kg/m^2^	12 (55)
Average systolic blood pressure ≥ 140 mmHg	9 (41)
	
Smoking-status	
Never-smoker	12 (55)
Ex-smoker	10 (45)
	
Null polymorphism of *GSTM1*^a^	10 (45)^b^

^a^*GSTM1*: Glutathione S-transferase M1

^b^Four subjects declined permission for genetic testing.

**Table 5:** Description of the study population clinical characteristics: current non-smoking subjects with type 2 diabetes mellitus.

Clinical Characteristics	N = 22 individuals
*Disease history*	Total number N (%) or mean (± SD)
Type 2 diabetes mellitus	22 (100)
Time since diabetes diagnosis [yrs]	6.4 (± 5.0)
Hyperlipidemia	19 (86)
Hypertension	19 (86)
Past myocardial infarction	0 (0)
Coronary artery disease	4 (18)
Peripheral vascular disease	3 (14)
Cerebrovascular disease	1 (5)
Diabetic retinopathy	1 (5)
Diabetic nephropathy^a^	8 (36)
	
*Blood marker levels*	Total number N (%)
Glycosylated hemoglobin A1c (HbA1c) ≥ 7%^b^	9 (41)^c^
Myeloperoxidase (MPO) ≥ 7 ng/ml^b^	41 (51)^d^
Ferritin ≥ 60 ng/ml^b^	36 (41)^e^
Adiponectin < 3700 ng/ml^b^	11 (50)^f^
Homocysteine ≥ 12 μmol/l^b^	11 (50)^c^
	
*Medication intake*	Total number N (%)
Sulfonylureas	10 (45)
Thiazolidinediones	6 (27)
Metformin	14 (64)
Statins	12 (55)
Aspirin	14 (64)
Beta-adrenergic receptor blockers (BB)	9 (41)
Angiotensin converting enzyme-inhibitors	12 (55)
Calcium-blockers	2 (9)
Diuretics	8 (36)
Angiotension II-receptor blocker	3 (14)
Estrogen	2 (9)

^a^Based on the screening urine (>30 μg albumin/mg creatinine) on spot collection

^b^Glycosylated hemoglobin A1c was only measured once, myeloperoxidase, adiponectin and homocysteine were measured on all four visits. For the interaction analysis with myeloperoxidase all observations per patient were used as it showed high daily variation within individuals. For ferritin all observations per patient were used as there were some single missing observations - however, there was only low daily variation within individuals. For the interaction analysis with adiponectin the mean of the four measurements per individual was used. For homocyteine the mean of the four measurements per individual was calculated.

^c^Data was missing from two subjects.

^d^Data was missing for 8 observations.

^e^Data was missing for 14 observations.

^f^Data was missing from one subject.

### Air pollutant and meteorology measurements

The 24-hour PM_2.5 _values over the study period were generally below the U.S. NAAQS of 35 μg/m^3 ^and the mean over the study period was below annual average NAAQS of 15 μg/m^3 ^(see Additional File 1: Table S1). The median of the within-individual PM_2.5 _ranges was 7.9 μg/m^3^. Correlations between PM_2.5 _and meteorology parameters as well as the time-course of PM_2.5 _and air temperature during the study period can be found in Schneider et al. (2008) [17].

### Description of health outcomes

A description of the patient means for the analyzed blood and ECG markers can be found in the Additional File 1: Tables S2-4. The majority of the blood parameters exhibited only low to moderate correlations. The medians of the patient-specific Spearman correlation coefficients within repolarization as well as HRV parameters of the 5-minute ECG were also on average low to moderate. HRV parameters of the 24-hour ECG correlated moderately to highly. As expected, BPsys and BPdia showed a high correlation. Correlation tables for all health outcomes can be found in the additional file (see Additional File 1: Tables S5-9).

### Association of PM2.5 with health outcomes

#### Blood parameters

**Figure 1:**
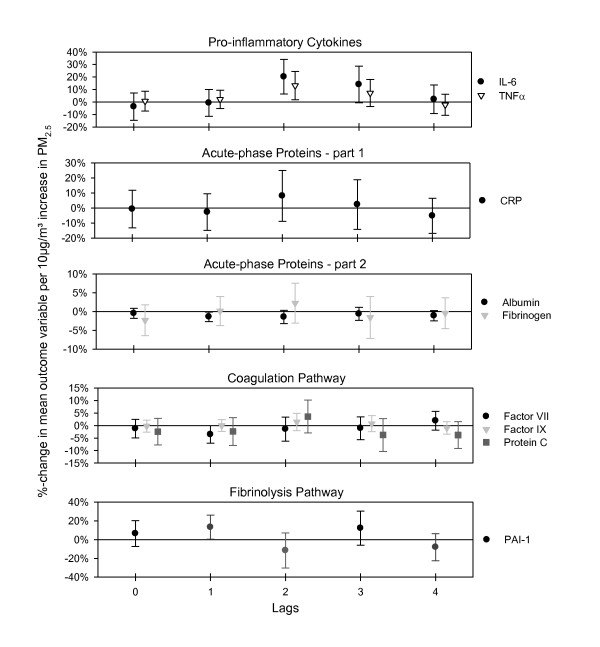
**Effect estimates for soluble blood-data with 95%-confidence intervals for immediate and delayed associations with PM_2.5_**.

The pro-inflammatory cytokines IL-6 and TNFα showed a strong increment in association with an increment in PM_2.5 _with a lag of two days, which persisted for one more day and then fell back to null (Figure 1). The acute-phase reactants CRP and fibrinogen did not exhibit as strong a response, but did increase slightly, however non-significantly, with a lag of two days. Albumin, a negative acute-phase protein, showed a decrease with a lag of one and two days. No associations with PM_2.5 _were found with blood parameters representing endothelial function and platelet activation (vWf, sICAM-1, sVCAM-1 and E-selectin) (data not shown). Markers of the coagulation pathway also showed slight, non-significant changes in association with PM_2.5_. In addition to the increase of fibrinogen with a lag of two days, protein C also increased slightly with the same time lag whereas factor IX remained unchanged. In contrast, factor VII showed a slight decrease after one day and also fibrinogen showed a decreasing tendency with a lag of 3 days (Figure 1). The fibrinolysis pathway marker PAI-1 increased with a lag of one day (Figure 1). Plasminogen, D-dimer (a fibrin degeneration product) and t-PA were not associated with PM_2.5_(data not shown).

**Figure 2:**
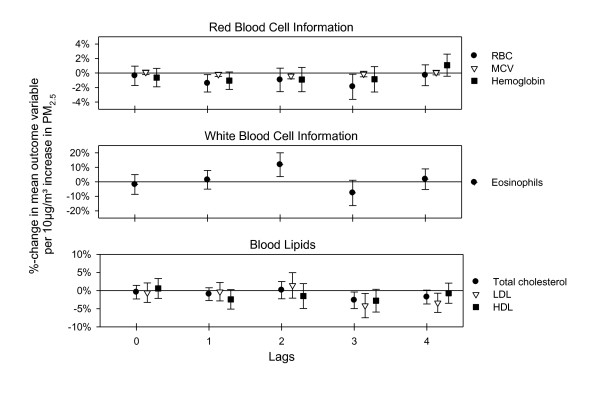
**Effect estimates for blood panel-data with 95%-confidence intervals for immediate and delayed associations with PM_2.5_**.

Decrements in total cholesterol (TC), high density lipoprotein (HDL) and low-density lipoprotein (LDL) were associated with PM exposure with a lag of three to four days (Figure 2). Red blood cells (RBC) showed a decrement with a lag of one to three days. Consistent with the RBC associations, mean corpuscular volume (MCV) and total hemoglobin also had decrements with a similar lag (Figure 2). Circulating eosinophils (absolute number and % of WBC) were positively associated with PM_2.5 _with a lag of two days, followed by a decrement at lag 3 (Figure 2). None of the other blood panel markers showed meaningful associations with PM_2.5 _(data not shown).

#### ECG parameters and blood pressure

**Figure 3:**
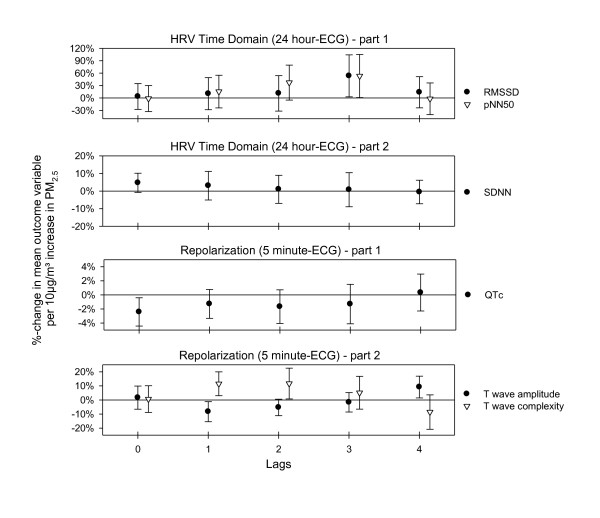
**Effect estimates for ECG-data with 95%-confidence intervals for immediate and delayed associations with PM_2.5_**. All estimates are shown for individuals without beta-blocker intake. For T wave amplitude one individual was excluded due to very strong associations at lag 0. For 24 hour-ECGs lag 0 means the association between the 24 hour air pollution average concurrent to the ECG measurement.

Analysis of ECG markers included an interaction term for the intake of beta-blockers, since it is known that they can alter ECG response to air pollutants. In the 5-minute resting ECGs, we did not find any association between heart rate or frequency domain parameters of HRV PM_2.5_. The 24-hour ECG time domain parameters showed an association with PM_2.5_; pNN50 increased with a lag of 2 and 3 days, and RMSSD increased with a lag of 3 days. SDNN showed a slight immediate increase for the non-beta-blocker subgroup (Figure 3).

QTc, the QT interval corrected for heart rate, decreased in association with air pollution at lag 0; and T wave complexity, a still relatively new marker to study ventricular repolarization, increased with a lag of 1 and 2 days for participants without beta-blocker intake (Figure 3). T wave amplitude, a marker for the morphology of the T wave, showed a strong association with PM_2.5 _in individuals not on beta-blockers: a decrease starting at lag 0 and persisting for lag 1 and 2 and ending with an increase at lag 4 (data not shown). Exclusion of one patient (female, Caucasian, 63 years, BMI = 20 kg/m^2^, taking diuretics and estrogens) who showed a very strong association with PM_2.5 _at lag 0 in the random slopes analysis, resulted in a loss in the lag 0-association, but led to much stronger effects for lag 1 and lag 2 and a weaker increase for lag 4 (Figure 3). The other repolarization markers (QT norm, QTVI, variability of T wave complexity) did not show any association with air pollution.

BPsys showed an immediate decrease at lag 0 (-2.7% [-5.5%; 0.1%]) and together with BPdia an increase with a delay of four days (BPsys: 2.6% [0.0%; 5.3%]; BPdia: 2.6% [0.2%; 5.0%]) in association with ambient PM_2.5_.

### Effect modification

**Figure 4:**
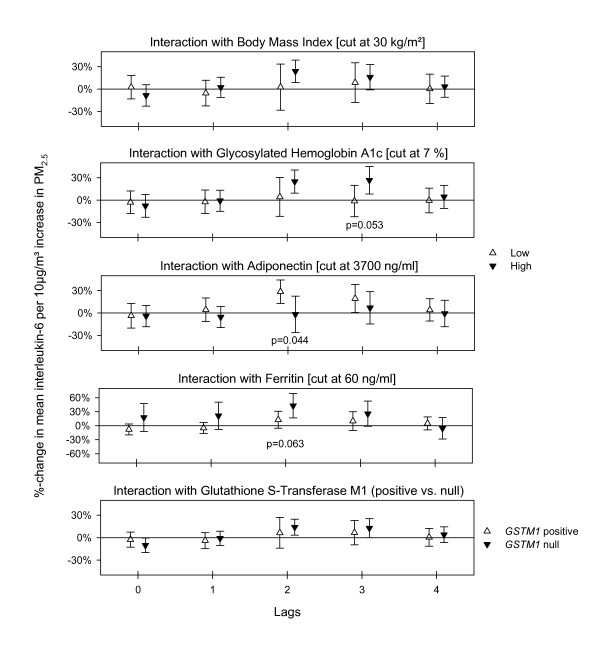
**Effect modification estimates for interleukin-6 with 95%-confidence intervals for immediate and delayed associations with PM_2.5_**. P-values for the significance of the interaction term are given if p ≤ 0.10.

**Figure 5:**
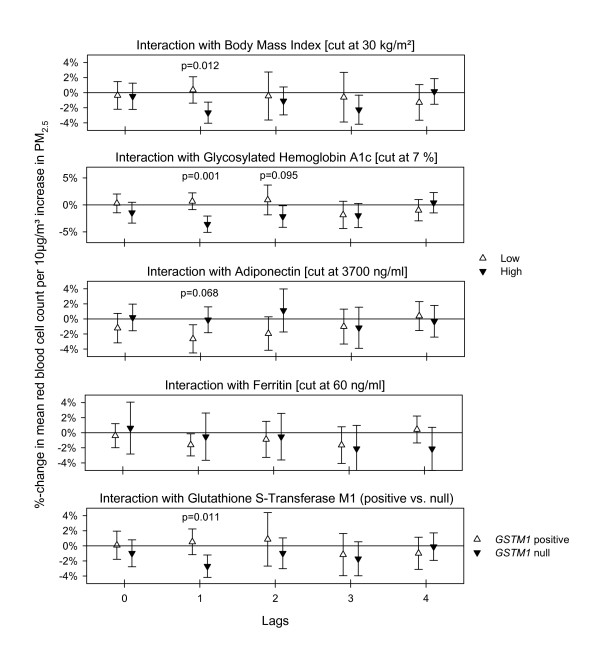
**Effect modification estimates for red blood cell count with 95%-confidence intervals for immediate and delayed associations with PM_2.5_**. P-values for the significance of the interaction term are given if p ≤ 0.10.

Interaction analysis for the inflammatory cytokine IL-6 revealed that stronger associations were observed in the subgroup with higher BMI, higher HbA1c value, and lower adiponectin levels, all markers associated with insulin resistance. Additionally, stronger associations were observed in those individuals with higher blood ferritin levels as well those carrying the *GSTM1 *deletion on both alleles (Figure 4). However, associations between TNFα and PM_2.5 _were strongest in the subgroup with lower BMI, lower HbA1c value, higher adiponectin level, and with at least one functional *GSTM1 *allele. Both cytokines showed a similar effect modification by ferritin (see Additional File 1: Figure S1). The decrease in factor VII was found mainly in the subgroup of participants with high BMI and high HbA1c (data not shown). Decreases in albumin were mainly found in individuals with higher BMI, higher HbA1c and lower adiponectin level (see Additional File 1: Figure S2). The interaction analysis with aspirin revealed only for protein C that the lag 2 response was much more pronounced for individuals without aspirin intake. Associations between PM_2.5 _and RBC or total hemoglobin were strongest in the subgroup with high BMI, high HBA1c, low adiponectin and the null *GSTM1 *genotype, as was seen for IL-6 (Figure 5 and Additional File 1: Figure S3). Eosinophils (absolute number count as well as % of WBC) increased slightly more at lag 2 in the subgroup of elevated BMI and HbA1c (data not shown). In contrast to our previously published work [17], interaction analysis with MPO levels did not show any notable effect modifications. No appreciable effect modification by age or by statins intake was found.

Since the models for the ECG marker analysis included an interaction term for the intake of beta-blockers, further effect modification analysis was not possible due to small sample size.

### Sensitivity analyses

Repeating the analysis without imputed PM values showed that the effect estimates didn't change appreciably. Health effects were observed below the current U.S. NAAQS 24-hour standard for PM_2.5 _as reducing the dataset to individuals that never experienced a day above 35 μg/m^3 ^during our relevant exposure period did not change the estimated effects. Changing the analyzed exposure from PM_2.5 _values measured on the roof of the EPHD in Chapel Hill, NC, to values obtained from fixed site community monitors resulted in similar effect estimates for the different health outcomes. The estimated effects were not sensitive to changing the covariance matrix from compound symmetry to first-order autocorrelation. Random slopes showed consistent PM_2.5 _associations across all study participants for IL-6 and TNFα (see Additional File 1: Figure S4), QTc, T wave amplitude, and T wave complexity (data not shown).

## Discussion

**Figure 6:**
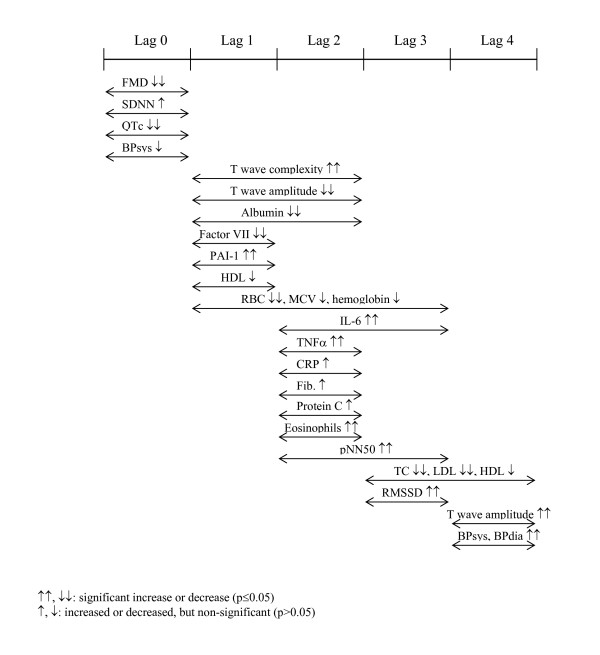
**Timing of health effects in association with ambient PM_2.5_**.

In this study we reported associations between PM_2.5 _levels and increased markers of systemic inflammation and hemostasis/thrombosis in people with diabetes. We also found PM-associated changes in autonomic nervous system control of heart rate as well as changes in ventricular repolarization. These changes did not occur simultaneously, but rather as a wave of response over a 4-5 day period (Figure 6). As previously reported [17] there is an immediate change in endothelial function (flow-mediated dilatation); here we also observed rapid changes in some electrocardiographic parameters (SDNN, QTc), as well as systolic blood pressure. Other measures of repolarization (T wave complexity, T wave amplitude) and vascular factors associated with clotting/fibrinolysis (Factor VII, PAI-1) as well as red blood cell-associated indices (RBCs, MCV, hemoglobin) changed with a lag of 1-3 days. Strong increases in the pro-inflammatory cytokines IL-6 and TNFα as well as blood eosinophils were observed with a delay of two days. Changes in markers of clotting/fibrinolysis (Factor IX, Protein C, fibrinogen) as well as CRP also occurred with the same lag. Finally, additional markers of heart rate variability (pNN50, RMSSD) and blood lipids (TC, LDL, HDL) changed with a lag of 3-4 days. Effect modification analyses revealed that overall the effects were more pronounced in the subgroup with clinical characteristics associated with insulin resistance (high BMI, high HbA1c, and low adiponectin level). Characteristics associated with greater oxidative stress potential (high ferritin level and *GSTM1 *null) also influenced PM associations found for IL-6 and RBC. It is unclear whether the immediate effects in endothelial function and electrocardiographic parameters are causally related to the more delayed effects in blood parameters or whether they occur via independent mechanisms.

### Inflammation and coagulation blood parameters

One of the more robust findings of this study is the association between PM and increases in TNFα and IL-6 levels in the blood. IL-6 acts as both a pro- and anti-inflammatory cytokine and plays a role in the acute phase response. TNFα plays a key role in the initiation of systemic inflammation and regulation of immune cells. The fact that both of these markers of inflammation are elevated in association with PM concentrations, as well as blood eosinophils, CRP, and fibrinogen (the latter both not significantly) indicates that ambient levels of PM are capable of inducing an inflammatory response in the blood.

Dubowsky et al.(2006) [11] also reported PM-associated increases in IL-6 and CRP in individuals with diabetes, with significance being achieved with a 3-4 day moving average of PM. Interestingly, in our study inflammatory markers were observed with a lag of two days. The effect size of the slight, but non-significant increases in CRP and fibrinogen with lag 2 in our study is in agreement with other air pollution epidemiological studies [14,15]. Our albumin results should be interpreted cautiously as albumin is often used as a measure of blood density and is an abundant blood protein that normally does not vary much. However, it can also be interpreted as an anti-acute phase protein and therefore the decreases at lag 1 and lag 2 fit well within the blood marker interplay of an inflammatory reaction. The strong delayed increase in eosinophils, an inflammatory leukocyte cell type, adds to this picture. However, the small increase in eosinophils in our non-asthmatic individuals is of uncertain significance regarding the inflammatory status and may also be related to an allergic or Th2 immune response governed by interleukin-5.

### Erythrocytes

We observed a decrease in RBC with a lag of 1-3 days, as well as decreased levels of hemoglobin and MCV. These findings are consistent with those of Seaton et al. (1999) [24] and Rückerl et al. (2007) [25], who saw significant decreases in both parameters in a similar time-frame. They and others have speculated that PM may cause constriction of small blood vessels or alter the adhesive properties of RBC, thus increasing the time these cells spend in systemic capillaries and not in larger vessels. Since increased oxidative stress is known to decrease the life span of RBC, it is also possible that PM-associated increases in oxidative stress which have been reported by many, could also result in fewer RBCs or could, perhaps, injure these sensitive cells causing leakage of hemoglobin and decreased volume.

### Blood lipids

In this study we observed an early decrease in HDL followed by decreases in HDL, LDL, and total cholesterol with a lag of 3-4 days. This is in contrast to an earlier study in which we observed increased levels of triglycerides and LDL associated with exposure of asthmatics to coarse PM [26]. However, it is in partial agreement with a study in which we reported decreased HDL/LDL ratio, VLDL, and triglycerides in healthy volunteers exposed to ultrafine particles [19]. One explanation for a PM-associated decrease in blood lipids is that PM-induced oxidative stress might modify blood lipids, making them more prone to adhere to vascular walls and removing them from the circulation. Kunitomo et al. (2009) [27] reported that exposure of mice to tobacco smoke resulted in oxidatively modified LDL. Araujo et al. [28] reported that exposure of mice to ultrafine and fine particles resulted in oxidized blood lipids as well as an inhibition of the anti-inflammatory capacity of HDL. Soares et al. (2009) [29] also reported that in mice exposed to urban air pollution LDL was oxidized and the lipid content of arterial plaques was increased. Another possible explanation for the observed lipid decreases is that they are related to changes known to occur during acute inflammation and the acute phase response. According to a review by Khovidhunkit et al. (2004) [30] infection and inflammation lead either to no change or to a decrease in serum cholesterol, LDL as well as HDL levels during the acute-phase response in humans.

Other studies have reported PM-associated changes in blood lipids. Suwa et al. (2002) [31] demonstrated in Watanabe heritable hyperlipidemic rabbits that exposure to PM_10 _increased the total amount of lipids in aortic lesions. Sun et al. (2005) [32] showed in a murine model that the lipid content in the aortic arch increased 1.5-fold in mice fed a high-fat chow diet and exposed to PM versus filtered air. In an epidemiologic case-control study of traffic-exposed police officers, the average values of HDL cholesterol and triglycerides were elevated in the exposed group versus an unexposed control group [33].

### ECG parameters and blood pressure

In our study, we found a slight immediate increase in SDNN and delayed positive associations between RMSSD, pNN50 and air pollution exposure, with the latter suggesting increased efferent vagal activity. Although many studies report a decrease in HRV associated with increasing PM concentration [7,8,34-38] and an exacerbation of these adverse PM effects in people with insulin resistance or diabetes [39,40], positive associations between PM and HRV have also been reported. An occupational study of NC State Highway Patrol troopers reported positive associations between PM and heart beat cycle length, HRV and ectopic beats on the next morning after their shift [41]. Wheeler et al. (2006) [42] reported a positive relationship between SDNN and PM_2.5 _in patients with pulmonary disease, whereas an inverse association was observed in patients with cardiovascular disease. Moreover, healthy [19] and asthmatic [43] volunteers exposed to concentrated ambient particles (CAPs) showed an increase in HF and LF approximately 18 hours after exposure.

In contrast to HRV, repolarization parameters have only recently been investigated in air pollution studies. In our study we observed an immediate negative association between PM_2.5 _exposure and QTc, indicative of vagal stimulation [44]. In addition, we observed changes in T wave amplitude. Decrements in T wave amplitude corresponded with increments in T wave complexity and vice versa. These findings are in agreement with other studies. In a study of male coronary artery disease patients, Henneberger et al. (2005) [45] reported an increase of QTc associated with organic carbon as well as a decrease of T wave amplitude and an increase of T wave complexity associated with ultrafine particles (UFP) and PM_2.5_. Frampton et al. (2004) [46] and Samet et al. (2009) [19] reported reduced QT intervals in healthy individuals breathing air and UFP with intermittent exercise. Zareba et al. (2009) [47] demonstrated small changes in QT interval, T wave amplitude and variability of T wave complexity in subjects exposed to UFP. We observed both early and late changes in T wave amplitude. It is possible that changes at earlier lags may be induced directly by changes via the autonomic nervous system or via changes in endothelial function (which was observed in our previous work [17] in the same study population), and changes at later lags may be triggered by inflammatory reactions.

Recent epidemiological and controlled exposure studies have reported small increases in BPsys and BPdia in association with PM at the same day as well as during a 5-day-average up to a 30-day-average [48,49]. The diabetic patients in our study showed an immediate decrease in BPsys and then a much delayed increase of BPsys and BPdia at a lag of 4 days. However, Ibald-Mulli et al. (2004) [50] also found small significant decreases in systolic as well as diastolic blood pressure in association with PM. We have not observed changes in blood pressure in studies in which volunteers were exposed to CAPs [51] and Brook et al. (2002) [52] did not observe changes in blood pressure in healthy subjects in a controlled exposure study with inhalation of fine CAPs and ozone. However, Urch et al. [53] reported an early increase in BPdia after controlled exposure to CAPs and ozone with the magnitude of the BPdia change being associated with the PM_2.5 _carbon content.

### Effect modification

Studies [11,54,55] have shown that patient characteristics such as high BMI or diseases like diabetes or hypertension increase the associations between PM and health outcomes. Obesity represents a pro-inflammatory state and as such may increase susceptibility to air pollution by increasing the response to inflammatory stimuli. We previously reported that markers of insulin resistance such as obesity and poor glycemic control as indicated by elevated HbA1c and low adiponectin were associated with a larger PM-associated decrease in endothelial dysfunction [17]. Here we show a similar finding for IL-6, RBC and total hemoglobin. Somewhat surprisingly, we observed that, while these patient characteristics were associated with increased PM-associated levels of IL-6, TNFα was increased more in those patients with lower BMI and better insulin control. Although both of these cytokines are associated with inflammation, they do not always travel together. They can be secreted by different cells under different control mechanisms and for different purposes. Blood levels of IL-6, but not TNFα have also been reported to be an independent predictor of type 2 diabetes after adjustment for BMI [56].

We observed that individuals with elevated levels of blood ferritin had increased levels of IL-6 and TNFα associated with PM exposure. Serum ferritin is an iron storage protein and considered a surrogate marker of total body iron levels. Higher levels of iron, often found in diabetic individuals, are hypothesized to make one more prone to inflammation and also to increase oxidative stress [57]. Excessive absorption and storage of dietary iron is also thought to contribute to the pathogenesis of insulin resistance, type 2 diabetes and its complications [58,59]. Ghio et al. (2008) [60] reported that inhaled tobacco smoke particles were able to mobilize endogenous iron present in lung lining fluid, resulting in altered iron homeostasis, increased metal-dependent oxidative stress, and ultimately increased levels of pulmonary inflammation. It is possible that patients with higher ferritin-levels would have more iron available for complexing with any particles that enter the vascular system and would thus have more oxidative stress leading to higher levels of inflammatory markers. Consistent with this hypothesis, in our study individuals with ferritin levels above 60 ng/ml showed much stronger increases in IL-6 and TNFα than those with lower ferritin levels.

Glutathione-S-transferase is a phase II enzyme which can scavenge oxygen-free radicals, metabolize reactive oxygen species and detoxify xenobiotics present in PM. People with a *GSTM1 *null allele are thought to handle oxidative stress less effectively and may be more responsive to agents such as PM which increase oxidative stress. The potential of this genotype to make carriers more susceptible to PM exposure has already been shown repeatedly [55,61-64]. We reported earlier that individuals with diabetes with the null *GSTM1 *polymorphism have the greatest PM-associated decreases in endothelial dysfunction. Here, we extend these findings by showing that individuals with diabetes carrying the null allele for *GSTM1 *also have the most pronounced changes in IL-6, RBC, total hemoglobin and MCV.

### Strengths and limitations

Excellent adherence to the protocol by the volunteers who attended all clinical visits is one of the study's strengths. Yet, the design with four successive visits in one week might not be optimal to detect changes in biomarkers having longer half-lives, such as fibrinogen. However, using first order autocorrelation as a covariance matrix to account for that autocorrelation between the data from consecutive days did not show strong influence on the effect estimation. Daily variations could have an influence on the health outcomes but models were adjusted for day of the week if AIC proved it to be necessary. Circadian variation was controlled by design, as measurements on every individual were taken at the same time of day.

A limitation of the study is the small number of individuals studied. While the numbers are adequate to observe associations between PM and pro-inflammatory cytokines, there was not enough power to exclude significant changes in other biomarkers related to inflammation and coagulation where only trends appeared. A major strength of the study was the simultaneous measurement of interacting ECG and blood markers, thereby establishing the temporal relationship of changes in physiological and biochemical responses to PM_2.5 _exposure, and providing insight into the potential mechanisms of air pollution effects. However, coagulation factors were measured as antigen levels, which don't necessarily reflect functional levels. As with any study that examines many different end points, multiple testing and as a consequence the potential for false-positive results can be a problem, especially with so many effect modifiers.

In this study we used daily PM_2.5 _mass values from a monitoring station located on the rooftop of the EPHD. In addition, PM_2.5 _values from a state monitoring station located 27 miles away from the EPHD were obtained. There was a strong correlation between these two sets of measurements, suggesting that we were accurately assessing ambient exposure of the study participants all living in a 30 mile radius of Chapel Hill. Thus, while ambient exposures are measured with some imprecision, these exposure measurement errors are non-differential with respect to either true ambient exposure or to the clinical measurements. As such, these Berkeson measurement errors will tend to bias our results towards the null. However, we have to acknowledge the possibility that the reported effect estimates might be modified by co-pollutants that were not measured. A further strength of this study is the investigation of air pollution effects in a particularly susceptible subgroup. On the other hand, the results cannot be generalized to the whole population.

## Conclusion

Exposure to elevated levels of ambient fine particulate air pollution showed a cascade of responses in association with PM_2.5_. PM_2.5 _might immediately alter ventricular repolarization and increase myocardial vulnerability to arrhythmias, as well as induce systemic inflammation two days later suggesting that some mechanisms work quickly through physiological systems primed to respond quickly, while some require a greater length of time for the activated signaling pathways to translate the response. It is not clear whether the mechanisms are causally related or independent. This study provides further evidence that individuals with diabetes appear to be very sensitive to PM_2.5_, especially those with higher insulin resistance. This issue is elevated to a higher level of concern given that the prevalence of diabetes is likely to increase over the next two decades.

## Competing interests

The authors declare that they have no competing interests.

## Authors' contributions

AS performed the statistical analyses and drafted the manuscript; LMN was involved in the design planning of the study as well as the analyses and interpretation of the data and revised the manuscript critically; DWG performed the analyses of the ECG markers and reviewed the manuscript critically; MCH was involved in the acquisition of the patient characteristics and health outcome data and reviewed the manuscript critically; WEC was substantially involved in the design of the study and reviewed the manuscript critically; MTS performed the analyses of some of the blood markers as well as performed the genotyping; JBB was involved in the enrollment of the diabetic individuals and reviewed the manuscript critically; AP was involved in the analyses and interpretation of the data; RBD was substantially involved in the design of the study, interpretation of the data and reviewed the manuscript critically;

All authors have read and approved the final manuscript.

## Supplementary Material

Additional file 1 **Table S1: Description of PM_2.5 _and of meteorology parameters throughout the study period (19 Nov 2004 to 09 December 2005)**. **Table S2**: Description of the inflammation and coagulation blood parameters (descriptive statistics was calculated from patient means). **Table S3**: Description of the blood panel parameters (descriptive statistics was calculated from patient means). **Table S4**: Description of the ECG and blood pressure parameters (descriptive statistics was calculated from patient means). **Table S5**: Correlation table for short-term (5 min.) repolarization markers. **Table S6**: Correlation table for short-term (5 min.) HRV markers. **Table S7**: Correlation table for long-term (24 hrs.) HRV markers. **Table S8**: Correlation table for systolic and diastolic blood pressure. **Table S9**: Correlation table for blood markers. **Figure S1**: Effect modification estimates for tumor necrosis factor alpha with 95%-confidence intervals for immediate and delayed associations with PM_2.5_. P-values for the stratum difference are given if p ≤ 0.10. **Figure S2**: Effect modification estimates for albumin with 95%-confidence intervals for immediate and delayed associations with PM_2.5_. P-values for the stratum difference are given if p ≤ 0.10. **Figure S3**: Effect modification estimates for total hemoglobin with 95%-confidence intervals for immediate and delayed associations with PM_2.5_. P-values for the stratum difference are given if p ≤ 0.10. **Figure S4**: Subject-specific associations (random slopes) with a 10 μg/m^3 ^increment in PM_2.5 _(lag of 2 days) for interleukin-6 (IL-6) and tumor necrosis factor alpha (TNFα). Click here for file
