# Supplementary material for: Association of cardiac and vascular changes with ambient PM2.5 in diabetic individuals
Source: Part Fibre Toxicol. 2010 Jun 2;7:14. doi: 10.1186/1743-8977-7-14 (PMC2896918; doi:10.1186/1743-8977-7-14)
Supplement: Additional file 1 — Table S1: Description of PM2.5 and of meteorology parameters throughout the study period (19 Nov 2004 to 09 December 2005). Table S2: Description of the inflammation and coagulation blood parameters (descriptive statistics was calculated from patient means). Table S3: Description of the blood panel parameters (descriptive statistics was calculated from patient means). Table S4: Description of the ECG and blood pressure parameters (descriptive statistics was calculated from patient means). Table S5: Correlation table for short-term (5 min.) repolarization markers. Table S6: Correlation table for short-term (5 min.) HRV markers. Table S7: Correlation table for long-term (24 hrs.) HRV markers. Table S8: Correlation table for systolic and diastolic blood pressure. Table S9: Correlation table for blood markers. Figure S1: Effect modification estimates for tumor necrosis factor alpha with 95%-confidence intervals for immediate and delayed associations with PM2.5. P-values for the stratum difference are given if p ≤ 0.10. Figure S2: Effect modification estimates for albumin with 95%-confidence intervals for immediate and delayed associations with PM2.5. P-values for the stratum difference are given if p ≤ 0.10. Figure S3: Effect modification estimates for total hemoglobin with 95%-confidence intervals for immediate and delayed associations with PM2.5. P-values for the stratum difference are given if p ≤ 0.10. Figure S4: Subject-specific associations (random slopes) with a 10 μg/m3 increment in PM2.5 (lag of 2 days) for interleukin-6 (IL-6) and tumor necrosis factor alpha (TNFα). [file 1743-8977-7-14-S1.DOC]

**Additional Material Table S1. Description of PM_2.5_** and of meteorology parameters throughout the study period (19 Nov 2004 to 09 December 2005).

| **Parameter** | **N** | **Mean** | **SD^a^** | **Min.** | **Max.** |
| --- | --- | --- | --- | --- | --- |
| **Environmental Public Health Division Rooftop PM_2.5_^b^** | | | | | |
| PM_2.5_^e^ [µg/m³], imputed | 383 | 14.2 | 7.2 | 1.5 | 42.8 |
| PM_2.5_^e^ [µg/m³] | 302 | 14.3 | 7.5 | 1.5 | 42.8 |
| **Environmental Public Health Division Rooftop Meteorology** | | | | | |
| Air temperature [°C] | 385 | 15.9 | 8.5 | -6.5 | 31.5 |
| Relative humidity [%] | 385 | 62.5 | 16.6 | 25.1 | 97.7 |
| Barometric pressure [hPa] | 386 | 1001.2 | 6.5 | 981.4 | 1021.9 |

^a^SD: standard deviation

^b^PM_2.5_: particulate matter with a diameter <2.5µm (study period: 386 days)

**Additional Material Table S**2. Description of the inflammation and coagulation blood parameters (descriptive statistics was calculated from patient means).

| **Parameter** | **N^*^** | **Mean** | **SD^a^** | **Min.** | **Max.** |
| --- | --- | --- | --- | --- | --- |
| Interleukin (IL)-6 [pg/ml] | 80 | 3.4 | 2.2 | 1.3 | 9.0 |
| Tumor necrosis factor (TNF) α [pg/ml] | 80 | 1.7 | 0.9 | 0.8 | 4.7 |
| C-reactive protein (CRP) [µg/ml] | 80 | 3.1 | 3.0 | 0.1 | 11.4 |
| Albumin [g/dl] | 79 | 4.4 | 0.3 | 4.0 | 5.0 |
| Fibrinogen [mg/ml] | 80 | 4.3 | 1.1 | 2.5 | 6.7 |
| Von Willebrand factor (vWf) [% activity] | 80 | 106.7 | 14.1 | 82.7 | 130.8 |
| Soluble intercellular adhesion molecules (sICAM)-1 [ng/ml] | 80 | 186.4 | 191.1 | 61.8 | 794.1 |
| Soluble vascular cell adhesion molecules (sVCAM)-1 [ng/ml] | 80 | 836.8 | 156.5 | 584.2 | 1125.3 |
| Soluble endothelial-leukocyte adhesion molecule (E-selectin) [ng/ml] | 80 | 35.7 | 17.8 | 15.1 | 82.1 |
| Factor VII [ng/ml] | 80 | 173.4 | 32.7 | 126.1 | 239.3 |
| Factor IX [µg/ml] | 80 | 4.1 | 0.7 | 3.2 | 5.9 |
| Protein C [µg/ml] | 80 | 3.4 | 0.7 | 2.1 | 4.7 |
| Plasminogen [IU^b^/ml] | 80 | 1.1 | 0.1 | 0.9 | 1.3 |
| tissue plasminogen activator (tPA) [ng/ml] | 80 | 6.1 | 6.0 | 1.3 | 22.4 |
| plasminogen activator inhibitor (PAI)-1 [ng/ml] | 80 | 30.8 | 17.7 | 4.9 | 75.4 |
| D-Dimer [ng/ml] | 80 | 623.1 | 190.8 | 318.8 | 974.0 |

^*^22 patients with a maximum of 4 measurements each

^a^SD: standard deviation

^b^IU: International units

**Additional Material Table S**3. Description of the blood panel parameters (descriptive statistics was calculated from patient means).

| **Parameter** | **N^*^** | **Mean** | **SD^a^** | **Min.** | **Max.** |
| --- | --- | --- | --- | --- | --- |
| Total cholesterol [mg/dl] | 79 | 180.7 | 48.9 | 114.0 | 284.8 |
| Triglycerides [mg/dl] | 79 | 156.8 | 84.6 | 61.0 | 372.5 |
| High density lipoprotein (HDL) [mg/dl] | 79 | 50.2 | 13.0 | 30.8 | 78.7 |
| Low density lipoprotein (LDL) [mg/dl] | 78 | 99.3 | 38.0 | 51.8 | 193.3 |
| Very low density lipoprotein (VLDL) [mg/dl] | 78 | 31.1 | 16.5 | 12.3 | 74.5 |
| Platelets [10³/µl] |  | 256.8 | 59.1 | 170.0 | 375.3 |
| Red blood cells count (RBC) [10^6^/µl] | 80 | 4.7 | 0.3 | 4.3 | 5.1 |
| Mean cell volume (MCV) [fl] | 80 | 87.8 | 3.3 | 80.3 | 93.5 |
| Total hemoglobin [g/dl] | 80 | 13.9 | 0.9 | 11.8 | 15.3 |
| Mean corpuscular hemoglobin (MCH) [pg] | 80 | 29.9 | 1.4 | 26.3 | 32.6 |
| White blood cells count (WBC) [10³/µl] | 80 | 6.2 | 1.4 | 3.7 | 9.4 |
| Monocytes [% WBC] | 80 | 7.1 | 1.7 | 4.8 | 11.5 |
| Neutrophils [% WBC] | 80 | 55.7 | 8.0 | 33.8 | 70.0 |
| Eosinophils [% WBC] | 80 | 2.1 | 1.1 | 1.0 | 5.3 |
| Lymphocytes [% WBC] | 80 | 34.4 | 7.5 | 22.0 | 56.5 |
| Monocytes [10³/µl] | 80 | 0.4 | 0.1 | 0.2 | 0.7 |
| Neutrophils [10³/µl] | 80 | 3.5 | 0.9 | 1.9 | 5.0 |
| Eosinophils [10³/µl] | 80 | 0.1 | 0.1 | 0.1 | 0.3 |
| Lymphocytes [10³/µl] | 80 | 2.2 | 0.8 | 1.1 | 5.3 |
| Glycosylated hemoglobin A1c (HbA1c) [%] | 20 | 6.7 | 0.9 | 5.4 | 9.1 |
| Ferritin [ng/ml] | 74 | 75.6 | 54.9 | 5.3 | 184.5 |
| Myeloperoxidase (MPO) [ng/ml] | 80 | 9.8 | 7.3 | 5.0 | 38.7 |
| Adiponectin [ng/ml] | 78 | 4726.8 | 4067.2 | 519.9 | 14121.6 |
| Homocysteine [µmol/l] | 76 | 12.9 | 3.4 | 7.4 | 19.9 |

^*^22 patients with a maximum of 4 measurements each (HbA1c: only one measurement per patient)

^a^SD: standard deviation

^b^IU: International units

**Additional Material Table S4. Description of the ECG and blood pressure** parameters (descriptive statistics was calculated from patient means).

| **Parameter** | **N^*^** | **Mean** | **SD^a^** | **Min.** | **Max.** |
| --- | --- | --- | --- | --- | --- |
| Root mean square of successive differences (RMSSD) (24hrs) [ms] | 65 | 65.4 | 39.4 | 28.0 | 188.0 |
| SD^a^ of NN^b^ (SDNN) (24hrs) [ms] | 65 | 104.8 | 24.6 | 66.7 | 163.3 |
| Percentage of adjacent NN^b^-intervals which differ more than 50ms (pNN50) (24hrs) [%] | 65 | 5.9 | 4.9 | 0 | 18.3 |
| Heart rate (HR) (5min) [1/min] | 86 | 66.9 | 10.8 | 50.0 | 90.0 |
| Bazett-corrected QT (QTc) interval (5min) [ms] | 86 | 433.1 | 25.6 | 384.5 | 495.5 |
| Normalized QT variance (QT_norm) (5min) [none] | 85 | 7.2*10^-3^ | 12.5*10^-3^ | 0.1*10^-3^ | 52.3*10^-3^ |
| QT variability index (QTVI) (5min) [none] | 85 | -0.4 | 0.8 | -1.6 | 1.1 |
| T wave complexity (5 min) [%] | 70 | 126.5 | 38.8 | 66.5 | 193.5 |
| Variability of T wave complexity (5min) [%] | 70 | 22.2 | 13.5 | 8.8 | 54.5 |
| T wave amplitude (5min) [μV] (without ID 11) | 86 | 228.2 (229.6) | 104.5 (106.9) | 68.5 (68.5) | 395.6 (395.6) |
| Total power (TP) [ms²] | 86 | 3073.8 | 4212.2 | 101.3 | 15922.5 |
| Low frequency band (LF) (5min) [ms²] | 86 | 669.2 | 907.5 | 13.0 | 3427.3 |
| Normalized low frequency band (LF_norm) (5min) [n.u.]^c^ | 86 | 33.9 | 10.9 | 15.8 | 61.8 |
| High frequency band (HF) (5min) [ms²] | 86 | 1125.5 | 1743.1 | 11.8 | 6669.8 |
| Normalized high frequency band (HF_norm) (5min) [n.u.]^c^ | 86 | 45.2 | 13.1 | 9.3 | 63.3 |
| LF/HF-ratio (5min) [none] | 82 | 1.0 | 1.4 | 0.3 | 7.2 |
| Systolic blood pressure (BPsys) [mmHg] | 84 | 132.8 | 17.9 | 97.0 | 180.5 |
| Diastolic blood pressure (BPdia) [mmHg] | 84 | 78.0 | 10.5 | 61.8 | 96.0 |

^*^22 patients with a maximum of 4 measurements each (24h-ECG: only 3 measurements each; HbA1c: only one measurement per patient)

^a^SD: standard deviation

^b^NN: normal-to-normal

^c^n.u.: normalized units

**Additional Material Table S5: Correlation table for short-term (5 min.) repolarization markers.**

|  | QTc | QT_ norm | QTVI | T wave amplitude | T wave complexity | Variability of T wave complexity |
| --- | --- | --- | --- | --- | --- | --- |
| QTc | 1 | -0.11 | -0.29 | 0.49 | 0.10 | 0.13 |
| QT_norm |  | 1 | 0.86 | -0.14 | 0.49 | 0.84 |
| QTVI |  |  | 1 | -.016 | 0.15 | 0.11 |
| T wave amplitude |  |  |  | 1 | -0.35 | -0.55 |
| T wave complexity |  |  |  |  | 1 | 0.70 |
| Variability of T wave complexity |  |  |  |  |  | 1 |

**Additional Material Table S6: Correlation table for short-term (5 min.) HRV markers.**

|  | HR | TP | LF_norm | HF_norm | LF/HF-ratio |
| --- | --- | --- | --- | --- | --- |
| HR | 1 | -0.21 | 0.17 | -0.56 | 0.12 |
| TP |  | 1 | -0.12 | 0.01 | 0.05 |
| LF_norm |  |  | 1 | -0.77 | 0.97 |
| HF_norm |  |  |  | 1 | -0.19 |
| LF/HF-ratio |  |  |  |  | 1 |

**Additional Material Table S7: Correlation table for long-term (24 hrs.) HRV markers.**

|  | RMSSD | SDNN | pNN50 |
| --- | --- | --- | --- |
| RMSSD | 1 | -0.03 | 0.80 |
| SDNN |  | 1 | 0.66 |
| pNN50 |  |  | 1 |

**Additional Material Table S8: Correlation table for systolic and diastolic blood pressure.**

|  | BPsys | BPdia |
| --- | --- | --- |
| BPsys | 1 | 0.84 |
| BPdia |  | 1 |

**Marked green: correlations from 0.40 to 0.69**

**Marked yellow: correlations from 0.70 to 1.00**

**Additional Material Table S9: Correlation table for blood markers.**

|  | CRP | Albumin | Fib. | IL-6 | TNFα | vWF | sICAM-1 | sVCAM-1 | E-selec-  tin | F VII | F IX | Protein C | Plasmino-  gen | D-dimer | tPA | PAI-1 | TC | Trigl. | HDL | LDL | VLDL | Platelets | RBC | MCV | Hemo-globin | MCH | WBC | Monos  (%) | Lymphs  (%) | Neutros  (%) | Eos  (%) | Monos  (abs) | Lymphs  (abs) | Neutros  (abs) | Eos  (abs) |
| --- | --- | --- | --- | --- | --- | --- | --- | --- | --- | --- | --- | --- | --- | --- | --- | --- | --- | --- | --- | --- | --- | --- | --- | --- | --- | --- | --- | --- | --- | --- | --- | --- | --- | --- | --- |
| CRP | 1 | 0.13 | 0.24 | -0.11 | -0.03 | 0.05 | -0.05 | 0.26 | -0.02 | 0.07 | 0.49 | 0.19 | 0.26 | 0.35 | 0.42 | 0.09 | 0.03 | -0.46 | 0.29 | 0.41 | -0.42 | 0.00 | 0.09 | 0.38 | 0.09 | -0.16 | -0.27 | 0.16 | -0.03 | 0.03 | -0.22 | 0.11 | -0.21 | -0.15 | -0.07 |
| Albumin |  | 1 | 0.26 | 0.06 | 0.00 | -0.02 | 0.08 | 0.09 | 0.14 | 0.31 | 0.28 | 0.49 | 0.27 | 0.13 | 0.16 | 0.27 | 0.57 | -0.06 | 0.50 | 0.42 | -0.06 | 0.30 | 0.48 | -0.05 | 0.42 | -0.17 | 0.18 | -0.27 | -0.24 | 0.25 | 0.09 | 0.02 | 0.13 | 0.23 | 0.26 |
| Fib. |  |  | 1 | 0.11 | -0.01 | 0.25 | 0.33 | 0.29 | 0.40 | 0.03 | 0.23 | 0.02 | 0.83 | -0.49 | 0.17 | 0.09 | 0.47 | -0.28 | 0.38 | 0.39 | -0.24 | 0.32 | 0.36 | 0.27 | 0.29 | -0.11 | 0.39 | 0.03 | -0.27 | 0.24 | 0.20 | -0.10 | 0.14 | 0.33 | 0.25 |
| IL-6 |  |  |  | 1 | 0.30 | 0.01 | 0.37 | 0.19 | 0.18 | -0.06 | -0.20 | 0.32 | 0.12 | -0.08 | 0.04 | -0.25 | 0.15 | 0.10 | 0.00 | 0.33 | 0.01 | 0.00 | 0.17 | -0.29 | 0.11 | 0.28 | -0.11 | -0.21 | -0.17 | 0.04 | 0.33 | -0.17 | 0.06 | 0.05 | 0.52 |
| TNFα |  |  |  |  | 1 | 0.39 | -0.17 | 0.17 | 0.14 | 0.21 | 0.03 | -0.22 | 0.39 | -0.35 | -0.12 | -0.15 | -0.02 | -0.02 | -0.35 | 0.04 | 0.00 | 0.09 | 0.21 | -0.33 | 0.15 | 0.04 | 0.00 | -0.24 | -0.27 | 0.20 | 0.02 | -0.02 | -0.21 | 0.27 | 0.14 |
| vWF |  |  |  |  |  | 1 | -0.31 | -0.06 | -0.06 | 0.04 | 0.15 | -0.48 | 0.49 | 0.21 | -0.20 | 0.07 | -0.27 | -0.47 | -0.16 | -0.32 | -0.38 | 0.02 | 0.20 | 0.24 | 0.07 | -0.23 | 0.15 | -0.05 | -0.12 | 0.12 | -0.07 | 0.20 | -0.08 | 0.15 | 0.00 |
| sICAM-1 |  |  |  |  |  |  | 1 | 0.56 | 0.72 | 0.29 | 0.53 | 0.43 | 0.35 | 0.16 | -0.02 | 0.04 | 0.33 | 0.14 | 0.41 | 0.08 | 0.05 | 0.16 | 0.22 | 0.26 | 0.16 | 0.16 | 0.41 | 0.00 | -0.04 | 0.05 | 0.03 | 0.17 | 0.18 | 0.26 | 0.07 |
| sVCAM-1 |  |  |  |  |  |  |  | 1 | 0.56 | 0.25 | 0.40 | 0.21 | 0.01 | -0.10 | 0.54 | 0.18 | 0.12 | -0.20 | 0.09 | 0.34 | -0.19 | 0.20 | 0.33 | -0.05 | 0.26 | 0.00 | 0.28 | 0.00 | 0.05 | -0.02 | 0.15 | 0.12 | 0.08 | -0.02 | -0.22 |
| E-selectin |  |  |  |  |  |  |  |  | 1 | 0.45 | 0.24 | 0.47 | 0.07 | 0.08 | 0.16 | 0.24 | 0.17 | 0.31 | 0.32 | -0.21 | 0.17 | 0.02 | 0.69 | 0.23 | 0.26 | -0.10 | 0.44 | -0.03 | -0.20 | 0.27 | 0.00 | 0.26 | 0.16 | 0.40 | -0.10 |
| F VII |  |  |  |  |  |  |  |  |  | 1 | 0.44 | 0.41 | 0.24 | 0.40 | 0.01 | -0.08 | 0.19 | 0.45 | 0.01 | 0.10 | 0.27 | 0.33 | 0.63 | 0.13 | 0.49 | 0.13 | 0.38 | 0.14 | 0.06 | -0.09 | -0.05 | 0.66 | 0.33 | 0.18 | -0.06 |
| F IX |  |  |  |  |  |  |  |  |  |  | 1 | 0.44 | 0.51 | 0.09 | 0.37 | 0.31 | 0.40 | -0.19 | 0.39 | 0.25 | 0.01 | 0.21 | 0.48 | -0.14 | 0.31 | 0.00 | 0.24 | -0.08 | 0.11 | -0.08 | 0.04 | -0.17 | 0.43 | 0.19 | -0.36 |
| Protein C |  |  |  |  |  |  |  |  |  |  |  | 1 | 0.30 | 0.33 | -0.02 | 0.36 | 0.45 | 0.54 | 0.59 | 0.46 | 0.27 | 0.28 | 0.62 | -0.57 | 0.47 | 0.12 | 0.10 | -0.11 | -0.09 | 0.08 | 0.32 | -0.18 | 0.17 | 0.08 | 0.33 |
| Plasminogen |  |  |  |  |  |  |  |  |  |  |  |  | 1 | -0.30 | 0.13 | 0.18 | 0.34 | -0.01 | 0.24 | 0.15 | 0.04 | 0.36 | 0.41 | 0.21 | 0.30 | -0.04 | 0.32 | -0.19 | -0.05 | 0.00 | 0.31 | -0.03 | 0.20 | 0.19 | 0.31 |
| D-dimer |  |  |  |  |  |  |  |  |  |  |  |  |  | 1 | 0.17 | 0.04 | -0.03 | -0.02 | 0.46 | -0.23 | 0.04 | 0.11 | 0.36 | 0.60 | 0.28 | -0.06 | 0.22 | 0.06 | 0.04 | 0.00 | 0.21 | 0.21 | 0.15 | 0.13 | -0.20 |
| tPA |  |  |  |  |  |  |  |  |  |  |  |  |  |  | 1 | -0.02 | 0.22 | 0.20 | 0.27 | 0.47 | 0.19 | 0.11 | 0.13 | 0.18 | 0.10 | -0.03 | 0.25 | -0.22 | 0.07 | -0.01 | 0.23 | 0.20 | 0.21 | 0.25 | 0.04 |
| PAI-1 |  |  |  |  |  |  |  |  |  |  |  |  |  |  |  | 1 | 0.19 | -0.29 | 0.36 | -0.01 | -0.27 | -0.04 | 0.03 | 0.47 | -0.02 | -0.16 | -0.33 | -0.01 | -0.11 | 0.20 | 0.00 | -0.05 | -0.50 | -0.22 | 0.18 |
| TC |  |  |  |  |  |  |  |  |  |  |  |  |  |  |  |  | 1 | 0.11 | 0.40 | 0.79 | 0.11 | 0.39 | 0.37 | -0.22 | 0.35 | 0.03 | 0.25 | -0.10 | -0.17 | 0.11 | 0.47 | 0.04 | 0.23 | 0.19 | 0.49 |
| Trigl. |  |  |  |  |  |  |  |  |  |  |  |  |  |  |  |  |  | 1 | -0.08 | -0.42 | 1.0 | -0.06 | 0.24 | 0.16 | 0.10 | 0.08 | 0.25 | -0.01 | 0.10 | -0.01 | 0.06 | 0.08 | 0.31 | 0.22 | -0.05 |
| HDL |  |  |  |  |  |  |  |  |  |  |  |  |  |  |  |  |  |  | 1 | 0.49 | -0.14 | 0.31 | 0.45 | 0.19 | 0.33 | -0.10 | 0.48 | -0.21 | -0.19 | 0.20 | 0.07 | 0.00 | 0.27 | 0.37 | 0.24 |
| LDL |  |  |  |  |  |  |  |  |  |  |  |  |  |  |  |  |  |  |  | 1 | -0.34 | 0.19 | 0.23 | -0.65 | 0.19 | -0.02 | 0.02 | -0.06 | -0.25 | 0.13 | 0.55 | 0.22 | 0.17 | -0.02 | 0.71 |
| VLDL |  |  |  |  |  |  |  |  |  |  |  |  |  |  |  |  |  |  |  |  | 1 | -0.06 | 0.27 | 0.13 | 0.11 | 0.05 | 0.21 | -0.03 | 0.15 | -0.05 | 0.08 | 0.13 | 0.34 | 0.20 | -0.09 |
| Platelets |  |  |  |  |  |  |  |  |  |  |  |  |  |  |  |  |  |  |  |  |  | 1 | 0.53 | 0.04 | 0.57 | 0.07 | 0.44 | -0.08 | -0.07 | 0.02 | 0.38 | 0.09 | 0.29 | 0.32 | 0.28 |
| RBC |  |  |  |  |  |  |  |  |  |  |  |  |  |  |  |  |  |  |  |  |  |  | 1 | -0.07 | 0.90 | -0.08 | 0.38 | -0.06 | -0.23 | 0.21 | 0.28 | 0.32 | 0.27 | 0.27 | 0.17 |
| MCV |  |  |  |  |  |  |  |  |  |  |  |  |  |  |  |  |  |  |  |  |  |  |  | 1 | 0.00 | -0.10 | 0.16 | 0.05 | 0.08 | 0.00 | -0.53 | 0.25 | 0.19 | 0.27 | -0.54 |
| Hemoglobin |  |  |  |  |  |  |  |  |  |  |  |  |  |  |  |  |  |  |  |  |  |  |  |  | 1 | 0.19 | 0.36 | -0.05 | -0.15 | 0.11 | 0.30 | 0.15 | 0.25 | 0.36 | 0.21 |
| MCH |  |  |  |  |  |  |  |  |  |  |  |  |  |  |  |  |  |  |  |  |  |  |  |  |  | 1 | 0.09 | 0.00 | 0.02 | -0.08 | 0.16 | 0.13 | 0.03 | 0.09 | 0.23 |
| WBC |  |  |  |  |  |  |  |  |  |  |  |  |  |  |  |  |  |  |  |  |  |  |  |  |  |  | 1 | -0.07 | -0.25 | 0.21 | 0.11 | 0.55 | 0.74 | 0.92 | 0.74 |
| Monos (%) |  |  |  |  |  |  |  |  |  |  |  |  |  |  |  |  |  |  |  |  |  |  |  |  |  |  |  | 1 | 0.28 | -0.45 | -0.23 | 0.76 | 0.00 | -0.22 | -0.23 |
| Lymphs (%) |  |  |  |  |  |  |  |  |  |  |  |  |  |  |  |  |  |  |  |  |  |  |  |  |  |  |  |  | 1 | -0.92 | -0.26 | -0.15 | 0.44 | -0.60 | -0.46 |
| Neutros (%) |  |  |  |  |  |  |  |  |  |  |  |  |  |  |  |  |  |  |  |  |  |  |  |  |  |  |  |  |  | 1 | 0.09 | -0.06 | -0.43 | 0.60 | 0.30 |
| Eos (%) |  |  |  |  |  |  |  |  |  |  |  |  |  |  |  |  |  |  |  |  |  |  |  |  |  |  |  |  |  |  | 1 | -0.31 | -0.01 | 0.05 | 0.80 |
| Monos (abs) |  |  |  |  |  |  |  |  |  |  |  |  |  |  |  |  |  |  |  |  |  |  |  |  |  |  |  |  |  |  |  | 1 | 0.19 | 0.46 | 0.17 |
| Lymphs (abs) |  |  |  |  |  |  |  |  |  |  |  |  |  |  |  |  |  |  |  |  |  |  |  |  |  |  |  |  |  |  |  |  | 1 | 0.23 | 0.24 |
| Neutros (abs) |  |  |  |  |  |  |  |  |  |  |  |  |  |  |  |  |  |  |  |  |  |  |  |  |  |  |  |  |  |  |  |  |  | 1 | 0.67 |
| Eos (abs) |  |  |  |  |  |  |  |  |  |  |  |  |  |  |  |  |  |  |  |  |  |  |  |  |  |  |  |  |  |  |  |  |  |  | 1 |

**Marked green: correlations from 0.40 to 0.69**

**Marked yellow: correlations from 0.70 to 1.00**

**Additional Material Figure S1: Effect modification estimates for tumor necrosis factor alpha with 95%-confidence intervals for immediate and delayed associations with PM_2.5_. P-values for the stratum difference are given if p≤0.10.**

**Additional Material Figure S2: Effect modification estimates for albumin with 95%-confidence intervals for immediate and delayed associations with PM_2.5_. P-values for the stratum difference are given if p≤0.10.**

**Additional Material Figure S3: Effect modification estimates for total hemoglobin with 95%-confidence intervals for immediate and delayed associations with PM_2.5_. P-values for the stratum difference are given if p≤0.10.**

**Additional Material Figure S4: Subject-specific associations (random slopes) with a 10μg/m^3^ increment in PM_2.5_ (lag of 2 days) for interleukin-6 (IL-6) and tumor necrosis factor alpha (TNFα).**
